# Interactions between Cisplatin and Quercetin at Physiological and Hyperthermic Conditions on Cancer Cells In Vitro and In Vivo

**DOI:** 10.3390/molecules25143271

**Published:** 2020-07-17

**Authors:** Nada Oršolić, Dyana Odeh, Maja Jazvinšćak Jembrek, Jelena Knežević, Darko Kučan

**Affiliations:** 1 Division of Animal Physiology, Faculty of Science, University of Zagreb, Rooseveltov trg 6, HR-10000 Zagreb, Croatia; dyana.odeh@biol.pmf.hr; 2 Division of Molecular Medicine, Laboratory for Protein Dynamics, Ruđer Bošković Institute, Bijenička cesta 54, HR-10000 Zagreb, Croatia; Maja.Jazvinscak.Jembrek@irb.hr; 3 Division of Molecular Medicine, Laboratory for Advanced Genomics, Ruđer Bošković Institute, Bijenička cesta 54, HR-10000 Zagreb, Croatia; Jelena.Knezevic@irb.hr; 4 University Hospital Merkur, Zajčeva 19, HR-10000 Zagreb, Croatia; dkucan@gmail.com

**Keywords:** multimodal treatment, cisplatin, quercetin, bladder cancer cell lines, intracavitary drug administration, mice

## Abstract

Quercetin (QU), a hyperthermic sensitizer, when combined with cisplatin (CP) affects tumor growth. To determine the effects of QU and CP and their interactions, multimodal treatment in vitro and in vivo models under physiological and hyperthermic conditions was performed. In vitro, different sensitivity of T24 and UMUC human bladder cancer cells was observed after short-term exposure to QU (2 h) and CP (1 h). Effects of both compounds were investigated at low and high micromolar concentrations (1 and 50 µM, respectively) under both thermal conditions. QU acted in additive or synergistic manner in combination with CP between physiological condition and hyperthermia. As determined by 3-(4,5-dimethylthiazol2-yl)-2,5-diphenyltetrazolium bromide (MTT) assay, short-term application of QU and CP reduced cell viability. Clonal assay also indicated that combined treatment with QU and CP is lethal to bladder cancer cells in both conditions. In vivo, CP (5 or 10 mg kg^−1^) and QU (50 mg kg^−1^) acted synergistically with hyperthermia (43 °C) and inhibited tumor growth, activated immune effectors and increased mice survival. Our results demonstrate that combined treatment with CP and QU may increase death of tumor cells in physiological and hyperthermic conditions which could be clinically relevant in locoregional chemotherapy.

## 1. Introduction

Bladder cancer (BC) is a significant problem worldwide and one of the leading cause of death. American Cancer Society estimated 81,400 new cases of BC and approximately 17,980 deaths in 2020 [1]. From the economic point of view, BC is the most expensive cancer to treat as it frequently requires diagnostic procedures such as cystourethroscopy, urine cytology, and radiological imaging [2]. Numerous risk factors may be involved in the development of BC, including smoking, work-related exposure to aromatic amines and polyurethane products, hereditary genetic background, nutritional factors, certain medical conditions, long-term treatment with chemotherapy agents, such as cyclophosphamide or immunosuppressive drugs like glucocorticoids, age, sex, ethnicity, race, and socio-economic status. BC mortality varies in different countries. The highest rates are reported in European countries such as Denmark, UK, Belgium, and Italy, whereas the lowest rates are observed in Asian countries, including Japan, China, and Singapore [3,4]. Some epidemiological data show that populations in South-East Asia have a 4- to 10-fold lower incidence of, and death from BC, when compared with those in the USA. These geographic discrepancies related to the incidence of many tumor types may be associated with differences in diet and intake of nutrients, of which some may have chemopreventive properties [4,5].

Treatment of BC with the conventional chemotherapeutic regimens is often associated with serious side effects (a strong systemic toxicity and local irritation) [6], while the efficacy of available intravesical immunotherapy agents such as bacillus Calmette–Guerin is ~30–70% [7]. Accordingly, a novel and more effective therapeutic approach is urgently needed for the treatment of BC. Administration of intravesical or intraperitoneal hyperthermal chemotherapy (HIVEC or HIPEC) results in high locoregional drug concentration. With this approach, systemic toxicity is limited by peritoneal–plasma barrier and its duration is relatively short (from 30 min to 2 h) [8]. This form of therapy leads to an increased exposure of tumor cells to the drug and long-term survival of patients and mice-bearing tumor [9,10,11]. Hyperthermia increases chemotherapy penetration at the peritoneal surface and may increase the sensitivity of the cancer cells to chemotherapy by impairing DNA repair machinery [9]. Hyperthermia may also induce apoptosis, activate heat-shock proteins that activate immune-modulating natural killer cells, and inhibit angiogenesis [9].

It is known that cancers originating from organs in the peritoneal cavity (e.g., ovarian, pancreatic, colorectal, gastric, and liver) and extra-abdominal tumors originating from lymphoma, lung, and breast as well as a small number of unknown primary tumors account for approximately 250,000 new cancer cases annually in the USA [10]. Despite advanced techniques and surgical removal of a macroscopically visible tumor in the peritoneal cavity, the problem of microscopic tumor residues remains, as well as the escape of tumor cells into the peritoneal cavity during surgery of advanced tumors that form an iatrogenic tumor [11]. Malignant ascites is a sign of peritoneal carcinomatosis, the presence of malignant cells in the peritoneal cavity. In many types of cancer, ascites is a prognostic sign of advanced stage, with a survival rate of 11% for patients suffering from ascites for more than 6 months [11]. Targeting cancer cells with nontoxic therapeutic agents differ in the mechanisms of action, enhances the treatment efficacy and is reported to have an additive or supra-additive effect [9,11]. Experimental tumors are known to be of great importance for the study of various forms of therapy. Ehrlich ascites tumor (EAT), a consequence of spontaneous breast cancer developed in female mice, is one of the most commonly used and adequate models of research in this area. EAT is referred as an undifferentiated carcinoma and is originally hyperdiploid, has high transplantable capability, no-regression, rapid proliferation, 100% malignancy, and also does not express tumor-specific transplantation antigen (TSTA) [12]. In an effort to improve local tumor control, a multimodality treatment strategy including locoregional hyperthermia, chemotherapy, and immunomodulation with quercetin, seems to be very attractive for treatment of various tumors.

There are various hypotheses on reduced cancer risk in subjects who consume fruits and vegetables rich in polyphenols/flavonoids. The health-beneficial effects of flavonoids, such as quercetin (QU), could be attributed to anti-cancer, anti-oxidative, anti-inflammatory, and anti-proliferative activities that are associated with the inhibition of bioactive enzymes and induction of detoxification enzymes [9,13]. However, cellular effects of these polyphenolic compounds are poorly understood. Along with protecting cells from oxidative stress, flavonoids can promote apoptosis and genotoxicity of tumor cells through pro-oxidant activities [14,15]. In addition to the pro-oxidative action and the initiation of apoptosis, other anti-tumorigenic mechanisms of QU action include inhibition of glycolysis and enzyme synthesis, interaction with estrogen type II binding sites, and modulation of numerous molecular targets [16,17,18]. The antitumor activity of QU has been investigated in different types of cancers including breast, colon, gastric, leukemia, melanoma, head and neck, and bladder cells [13,16,17,18,19].

In addition to anti-tumor effects, QU acts as a hyperthermic sensitizer in HeLa cells, melanoma cells, and lymphoid-leukemia and ovarian cell lines, by inducing intracellular lactate accumulation and acidification. QU also inhibits tumor growth and amplifies the effects of hypothermia in PC-3 and DU-145 prostate tumor cells [20]. Furthermore, QU reduces temperature-dependent expression of heat-shock protein-70 (HSP-70) by downregulating heat-shock-transcription factor 1 (HSF-1) in vitro [21,22,23], and in vivo, as shown in colon carcinoma and Ehrlich ascites tumor (EAT) in mice [9,24]. Of note, several studies have demonstrated that hyperthermia may be useful in overcoming resistance to chemotherapeutic drugs such as CP by increased drug uptake, changing in the tumor microcirculation, blood flow, cell membrane permeability, and cellular metabolism [9,11,19]. CP is the most widely used chemotherapeutic with numerous side effects, including hepatorenal toxicity, cardiotoxicity, neurotoxicity, ototoxicity, and changes of hematological parameters [9]. It is applied intraperitoneally for bladder, cervical, ovarian cancer, gastric cancer, non–small cell lung cancer, mesothelioma, and head-and-neck squamous cell carcinoma. Cisplatin enters into cell by binding to DNA to form a DNA adduct that interferes with DNA transcription and replication. As a consequence, it abolishes the cell cycle, leading to activation of DNA repair mechanism and induction of apoptosis. In addition, CP can cause immunogenic cell death which is further enhanced by hyperthermia [9,11].

Therefore, QU has received much attention as a promising anticancer compound with little toxicity, but also as an adjuvant to the therapy capable to reduce CP-induced toxicity and sensitize cells to hyperthermia by antagonizing the induction of the heat shock response and ability to increase immunomodulatory activity [9,20]. However, it was also reported that low concentrations of QU may attenuate effects of CP and other anti-neoplastic drugs in ovarian cancer cells by reducing reactive oxygen species (ROS)-induced damage [25].

Moreover, some of the mechanisms of QU action are very poorly defined. In particular, the differences between its in vitro and in vivo activities, the efficacy of multimodal approach in the therapy of tumors in combination with chemotherapy and hyperthermia, mode of QU interactions with chemotherapeutics and hyperthermia, as well as advantages and disadvantages of QU application in different models.

Hence, to better understand the efficacy of the combined treatment with QU and CP and effects of QU/CP interactions on tumor growth, we performed a multimodal study in in vitro and in vivo systems under physiological and hyperthermic conditions, which mimic intraoperative hyperthermic intraperitoneal chemotherapy (HIPEC) condition when the tumor is in an advanced stage of growth. 

## 2. Results

**Figure 1:**
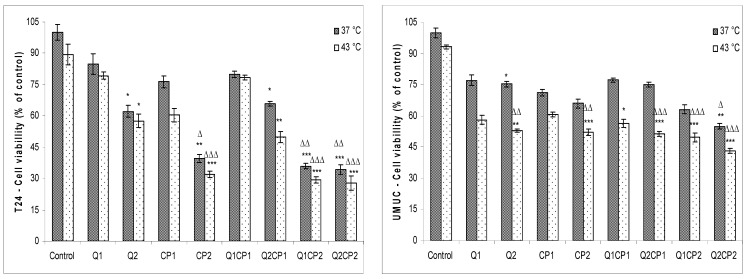
Effect of quercetin (QU), cisplatin (CP), and their combinations on the viability of T24 and UMUC human bladder cancer cells under physiological and hyperthermic conditions. T24 and UMUC cells were preincubated with 1 or 50 µM QU for 2 h at 37 °C, washed with phosphate-buffered saline (PBS) and incubated in fresh medium with or without 1 or 50 µM CP for 1 h under physiological and hyperthermic conditions. Cell viability was determined by 3-(4,5-dimethylthiazol2-yl)-2,5-diphenyltetrazolium bromide (MTT) assay. The data are expressed as mean ± SD of cell viability compared to control from three independently performed experiments. * Significantly different (* *p* < 0.05; ** *p* < 0.01; *** *p* < 0.01, nonparametric Kruskal–Wallis test) from control group 37 °C. ^Δ^ Significantly different (^Δ^ *p* < 0.05; ^ΔΔ^ *p* < 0.01; ^ΔΔΔ^ *p* < 0.01, nonparametric Kruskal–Wallis test) from control group 43 °C. Abbreviation: QU1 or QU2, treatments with quercetin at concentrations of 1 or 50 µM; CP1 or CP2, treatments with cisplatin at concentrations of 1 or 50 µM.

To examine the effects of QU and CP on T24 and UMUC human bladder cancer cells under physiological and hyperthermic conditions, exponentially growing cells were treated with 1 or 50 µM QU (QU1 or QU2) and incubated for 2 h, washed and treated with or without 1 or 50 µM CP (CP1 or CP2) for 1 h under physiological (37 °C) and hyperthermic conditions (43 °C), respectively. As determined by 3-(4,5-dimethylthiazol2-yl)-2,5-diphenyltetrazolium bromide (MTT) assay, treatment with QU and/or CP reduced viability in both cell lines, although higher sensitivity was observed for T24 cells (Figure 1).

**Figure 2:**
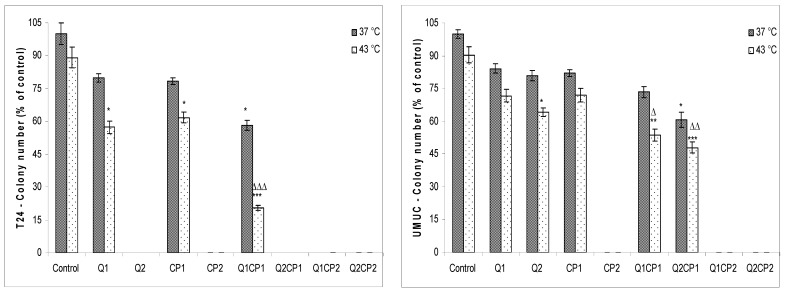
Colony formation efficiency of quercetin (QU), cisplatin (CP) and their combinations in T24 and UMUC human bladder cancer cells under physiological and hyperthermic conditions. T24 and UMUC cells were preincubated with 1 or 50 µM QU for 2 h at 37 °C, washed with phosphate-buffered saline (PBS) and incubated in fresh medium with or without 1 or 50 µM CP for 1 h under physiological and hyperthermic conditions. Following treatment with CP, cells were rinsed again with PBS for three times to remove the CP and afterwards were grown in incubator for up to 14 days in complete culture media. After 14 days, colonies were fixed with 100% methanol, stained with Giemsa stain and the plating efficiency (PE) was calculated as PE = (Colonies formed/Cells seeded) × 100%. The data are expressed as mean ± SD of colony formation efficiency in comparison to control from three independently performed experiments. *Significantly different (* *p* < 0.05; ** *p* < 0.01; *** *p* < 0.01, nonparametric Kruskal-Wallis test) from control group at 37 °C. ^Δ^ Significantly different (^Δ^ *p* < 0.05; ^ΔΔ^ *p* < 0.01; ^ΔΔΔ^ *p* < 0.001, nonparametric Kruskal-Wallis test) from control group at 43 °C. Abbreviations: QU1 or QU2, treatments with quercetin at concentrations of 1 or 50 µM; CP1 or CP2, treatments with cisplatin at concentrations of 1 or 50 µM.

Hyperthermia in both cell lines additionally reduced the survival rate up to 10% and caused very low sensitization to CP. Again, the effect was more pronounced in T24 than UMUC cell line (Figure 1). There were no significant differences in the percentage of cell viability (MTT test) under physiological and hyperthermic conditions for T24 cells treated with QU: percentage of cell viability for Q1 was 84.9 ± 4.97% at 37 °C vs. 79.3 ± 1.55% at 43 °C (*p* ˃ 0.05) and for Q2 was 62.2 ± 2.87% at 37 °C compared to 57.67 ± 3.14% at 43 °C (*p* ˃ 0.05). Treatment with CP reduced survival of T24 cells to 76.3 ± 2.89% (CP1) or 39.5 ± 1.98% (CP2) at 37 °C, in comparison to 60.4 ± 3.22% (CP1) or 32.1 ± 1.55% (CP2) at 43 °C. The combined treatment (QU1CP2 and QU2CP2) showed a significantly higher effect in relation to control under both condition (37 °C and 43 °C; *p* < 0.001), Q2 (*p* < 0.05), but not in comparison to CP2. There was no significant difference between the different thermal conditions (37 or 43 °C) in combined treatment. Similar data were obtained for the UMUC human bladder cell line but with lower sensitivity on combined treatment and the different thermal conditions and without differences between applied concentration of QU and CP (1 or 50 µM). Apart from the results obtained with MTT assay, QU and CP showed even higher ability to reduce cell clonogenesis (Figure 2).

Cell clonogenesity was significantly inhibited by hyperthermal treatment in both cell lines (Figure 2). These data indicated that tested compounds exerted a significant cytotoxic effect in higher concentration on both cell lines and effect was concentration-dependent in T24 cells. Combined treatment with CP and QU in all combinations, except for QU1CP1, had a lethal effect on T24 cells under both, physiological and hyperthermic conditions. It is also evident that treatments with Q2 and CP2 alone were lethal for T24 cells. Visual inspection of the plates 14 days after the treatment of UMUC cells, revealed no cells or colonies following exposure to low and high concentration of QU (QU1 and QU2) in the presence of CP2 (QU1CP2 and QU2CP2), under physiological and hyperthermic conditions. Higher concentration of CP also completely prevented colony formation irrespective of the temperature applied.

In order to verify whether the effect of the combined treatment of QU with CP at 37 or 43 °C was due to an antagonistic, additive or synergistic action, combination index values were calculated, as described in the section Materials and Methods and are presented in Table 1 and Table 2.

The combination indices (CI) were determined from the data obtained by MTT (Table 1) and clonogenic assays (Table 2). The obtained values of CI in MTT analysis for T24 cells suggest that low concentration QU (1 µM) in combination with CP in both concentration induce additive effect under physiological conditions while higher concentration of QU (50 µM) with CP induce synergistic effect under both, physiological and hypertermic conditions (Table 1). In contrast, both concentrations of QU in combination with CP exert a synergistic effect (Table 1, UMUC cells).

However, in clonogenic assay, combined effect of QU1CP1 under physiological and hyperthermic conditions may induce additive effect in T24 cells while all other combination were lethal for T24 cells because treatment with 50 µM CP or QU in both condition showed complete lack of cell viability and colony formation, and inability to repair (Table 2). UMUC cells were also very sensitive to higher concentration of CP (CP2); CP2 combination with QU was lethal to UMUC cells (Table 1) while combination QU in both concentration with CP1 induce additive effect in hyperthermic conditions.

### In Vivo Effects

As it is difficult to achieve a specific tumor temperature in the clinic because of the various types of heat conduction due to specific tissue vascularization, we investigated the local effects of QU and CP in vivo on the EAT cells. After disinfection of external abdominal region, each animal was inoculated with 3 mL of saline solution and after gentle agitation of abdominal wall, the solution containing peritoneal cells was removed for cellular evaluation. The following variables were analyzed: the total number of cells and differential count of the cells present in the peritoneal cavity, tumor inhibition (%) and functional activity of macrophages. The remaining animals (*n* = 9 of each group) were used for the survival analysis.

**Figure 3:**
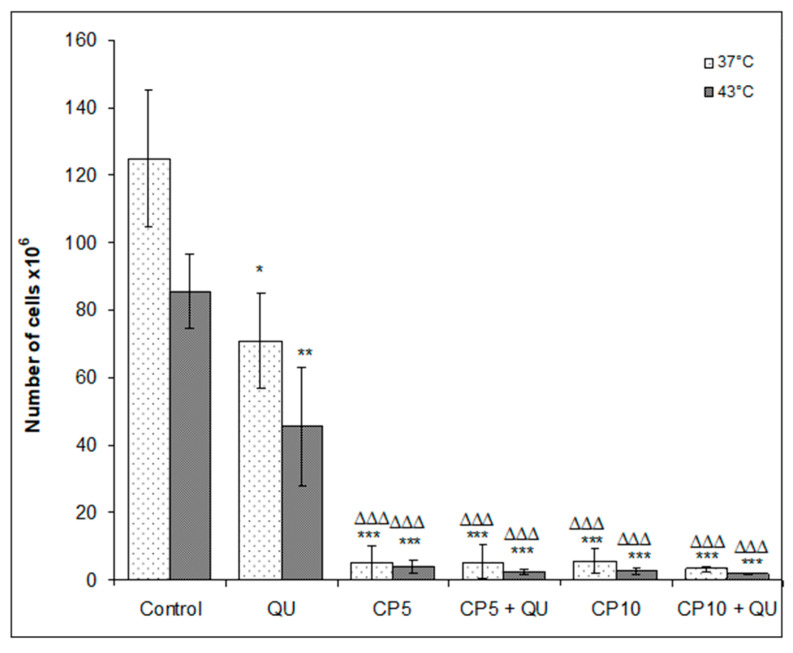
Total number of tumor cells in the intraperitoneal cavity of Swiss albino mice bearing Ehrlich ascites tumor after therapeutic treatment with cisplatin (CP) and quercetin (QU) under physiological and hyperthermic conditions. Mice were treated with CP (5 or 10 mg kg^−1^, *i.p.*) 5 days after *i.p.* inoculation of EAT cells (2 × 10^6^/mouse) under physiological and hyperthermic conditions and with QU (*i.p.* 50 mg kg^−1^) at day 6 and 12 after *i.p.* inoculation of EAT cells. Results are reported as the mean ± SD (*n* = 6). * Significantly different (nonparametric Kruskal–Wallis test, * *p* < 0.05; ** *p* < 0.01); *** *p* < 0.001) from control at 37 °C. ^Δ^Significantly different (^ΔΔΔ^ *p* < 0.001, nonparametric Kruskal–Wallis test) from control group at 43 °C. Abbreviation: EAT, Ehrlich ascites tumor; QU, treatment with quercetin at a dose of 50 mg kg^−1^; CP5 or CP10, treatment with cisplatin at doses of 5 or 10 mg kg^−1^.

Animals exposed to intraperitoneal hyperthermia had a significant decrease of the proliferative activity of EAT cells. Numbers of EAT cells in control groups under physiological and hyperthermic conditions were (124.88 ± 25.19) × 10^6^ and (85.5 ± 11.23) × 10^6^, (Figure 3), while mean survival time was 20.67 and 24.78 days, respectively (Table 3).

**Figure 4:**
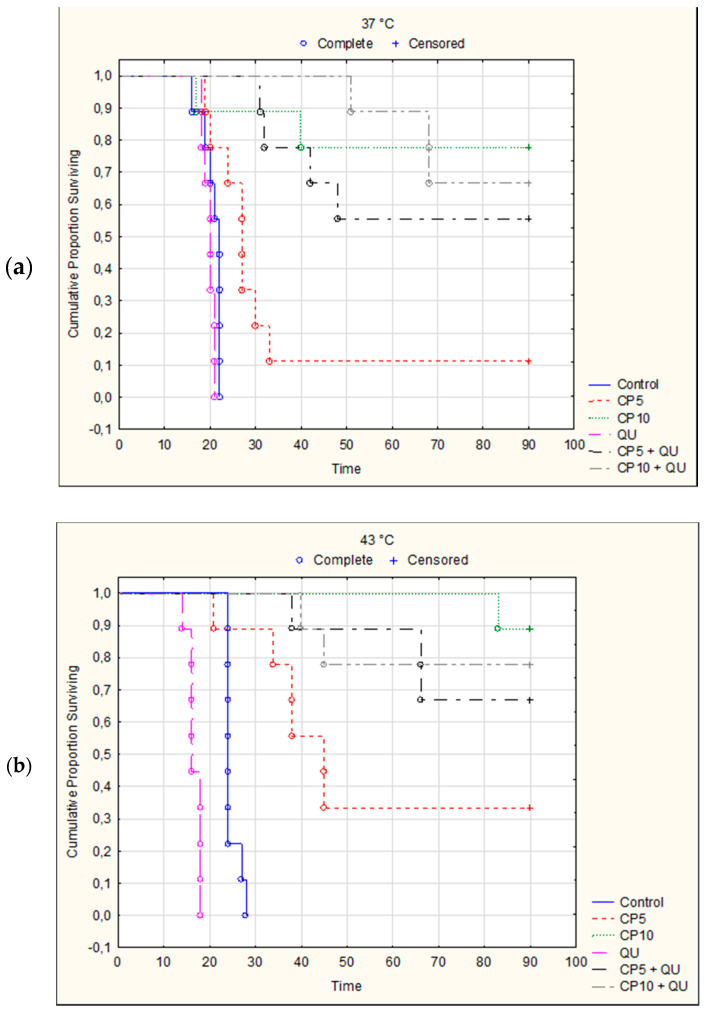
Kaplan–Meier survival curves of Swiss albino mice bearing Ehrlich ascites tumor after therapeutic treatment with quercetin and cisplatin under physiological condition (**a**) and hyperthermic condition (**b**). Mice were treated with CP (5 or 10 mg kg^−1^, *i.p.*) 5th day after *i.p.* inoculation of EAT cells (2 × 10^6^/mouse) under physiological and hyperthermic conditions and with QU (*i.p.* 50 mg kg^−1^) at day 6 and 12 after *i.p.* inoculation of EAT cells. The number of mice in each experimental group was 9. Statistical significance in animal survival under physiological or hyperthermic conditions and between controls under both temperature is shown in Table 4.

Treatment of mice with QU alone at a dose of 50 mg kg^−1^ reduced the number of cells in peritoneal cavity under both physiological and hyperthermic conditions (TI% was 43.27% and 64.39%), but was without the effect on the survival (Figure 4 and Table 3). The very pronounced antitumor effect was achieved after treatment with CP at doses of 5 or 10 mg kg^−1^ at 37 and 43 °C, and after treatment with CP in combination with QU (Figure 3, Table 3). TI% for CP5 or CP10 and its combination with QU were from 95.56 to 98.61%. In addition to significant reduction of tumor cell number compared to the control (*p* < 0.001), these groups also had a significant increase of lifespan (Table 3 and Figure 4).

**Figure 5:**
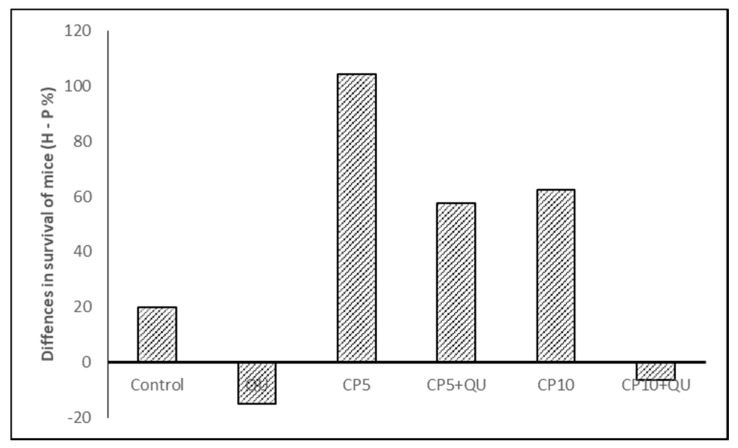
The difference in the percentage of survival of Swiss albino mice bearing Ehrlich ascites tumor after therapeutic treatment with cisplatin (CP) and quercetin (QU) within the hyperthermic conditions in relation to physiological conditions. Abbreviation: QU—treatments with quercetin at dose of 50 mg kg^−1^; CP5 or CP10—treatments with cisplatin at dose of 5 or 10 mg kg^−1^.

It is important to emphasize that significantly increased survival time was observed after treatment with CP at 37 °C and especially at 43 °C in relation to control group. CP at a dose of 5 mg kg^−1^ increased the life span (ILS) at 37 °C for 59.65% and for 163.96% at 43 °C. In the same group, one mouse exposed to physiological and three mice exposed to hyperthermic conditions were long term survivors (*p* = 0.0060; *p* = 0.0004, Kaplan–Meier analysis). ILS% of mice treated with CP at a dose of 10 mg kg^−1^ was 269.28 and 331.64 under physiological and hyperthermic conditions, respectively, whereas seven or eight mice were long term survivors (*p* = 0.0060; *p* < 0.0001, Kaplan–Meier analysis). Furthermore, significant increase of the survival time was observed after combined treatment with CP5 and QU at 37 °C, in relation to group treated only with CP. Values of ILS% in CP5 + QU group were 224.14 and 59.65, while in CP10 + QU group life span was increased by 290.81% and 269.28% when compared with control group inoculated with EAT cells at 37 °C. Five mice were long-time survivors in CP5 + QU treated group (*p* = 0.0001, Kaplan–Meier analysis), while six mice were long-time survivors in CP10 + QU group (*p* = 0.0001, Kaplan–Meier analysis). Interestingly, in hyperthermic conditions the difference between the observed increases of the survival in CP5 + QU and CP5 groups was 117.41% (ILS% for CP5 + QU was 281.36 vs. 163.96 in CP5 group), and 6 mice were long-time survivors in CP5 + QU group *(p* < 0.0001, Kaplan–Meier analysis). On the contrary, the lifespan in CP10 + QU group was decreased (the difference was negative, −47.32%) in relation to CP10 group (ILS% for CP10 + QU was 284.32 vs. 331.64 in CP10 group). The number of long-time survivors in CP10 and CP10 + QU groups was eight and seven, respectively (*p* < 0.0001). Of the nine mice in the CP10 + QU group, two mice died on day 40 and day 45 after implantation due to severe drug toxicity. More exactly, the difference in survival within hyperthermic conditions with respect to physiological conditions (hyperthermic minus physiological treatment) in all experimental groups was as follows: control, 19.88; QU, −15.04; CP5, 104.30; CP5 + QU, 57.52; CP10, 62.36, and CP10 + QU, −6.48, respectively (Figure 5). Despite the increase in survival rate, there was no statistically significant difference in survival between the corresponding groups treated under physiological conditions compared to hyperthermic conditions, except for treatment with cisplatin at a dose of 5 mg kg^−1^.

Combination indices in vivo were calculated from the data obtained by trypan blue (TB) exclusion assay. Analysis of the obtained values suggests that combined treatments CP5 + QU or CP10 + QU may induce synergistic effect (CI = 1.826 ± 0.20 or CI = 2.363 ± 0.13) under physiological and hyperthermic conditions (CI = 1.660 ± 0.23 or CI = 1.857 ± 0.31) (Table 5).

The analysis was made according to the data from Figure 4. The combination index was calculated as described in Materials and methods. The combined effects of QU or CP and HT (combination index) was calculated using the formula %AB/(%A × %B), where A and B are the effects of each individual agent and AB is the effect of the combination. *P*, statistical significance values of the combination indices (AB) compared with the additive combination index [AB/(A × B)], nonparametric Kruskal–Wallis test, *p* < 0.05. Results are reported as the mean ± SD (*n* = 6). Abbreviation: QU—treatment with quercetin at a dose of 50 mg kg^−1^; CP5 or CP10—treatments with cisplatin at doses of 5 or 10 mg kg^−1^.

In addition, during CP and hyperthermia treatment, no significant statistical differences were observed between control and experimental animals regarding behavior and food consumption. No weight loss, diarrhea, skin lesions or other specific abnormalities were noticed.

**Figure 6:**
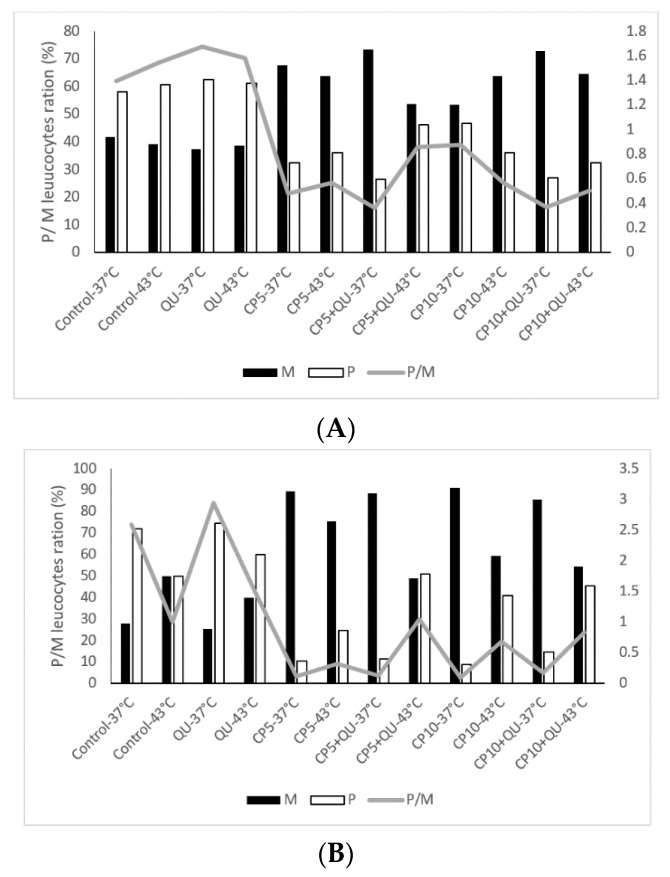
Changes in Polymorphonuclear/Mononuclear Leukocyte Ratio—P/M activity of Swiss albino mice bearing Ehrlich ascites tumor after therapeutic treatment with QU and cisplatin under physiological and hyperthermic conditions at day 13 (**A**) and day 20 (**B**). Mice were treated with CP (5 or 10 mg kg^−1^, *i.p.*) 5th day after *i.p.* inoculation of EAT cells (2 × 10^6^/mouse) under physiological and hyperthermic conditions and with QU (*i.p.* 50 mg kg^−1^) at day 6 and 12 after *i.p.* inoculation of EAT cells. The results are expressed as mean value of each experimental group (*n* = 6). Abbreviations: QU—treatment with quercetin at a dose of 50 mg kg^−1^; CP5 or CP10—treatments with cisplatin at doses of 5 or 10 mg kg^−1^; P—polymorph nuclear leukocytes; M—mononuclear leukocytes; P/M—ratio between polymorph nuclear leukocytes and mononuclear leukocytes.

It is known from the literature that many of the anticancer drugs, such as CP, are very toxic. The role of the flavonoids might be important in ameliorating their toxicity on liver, kidney, complete blood count and blood parameters abnormalities. Differential blood analysis clearly demonstrates that hyperthermia in combination with CP changed the ratio between the polymorph nuclear and mononuclear leukocytes (Figure 6A,B), which coincided with the number of active macrophages in the peritoneal cavity (Table 6). Macrophage spreading index was increased under hyperthermic conditions (Table 6).

Finally, hematological and blood parameters were monitored on day 13 and 20 after tumor inoculation. No significant abnormalities in renal (urea, creatinine) and liver biomarkers were observed at day 13 after treatment with CP at both doses (5 or 10 mg kg^−1^) or during combined treatment with QU (CP + QU). On day 20 after inoculation, we noted improvement in renal and hepatic function (AST, ALT, and LDH) in relation to control group and mice treated only with QU (data not shown), but observed changes did not reach the level of statistical significance (*p* > 0.05).

## 3. Discussion

According to previous research, temperatures above 42 °C have shown direct lethal effect on cancer cells in a time- and temperature-dependent manner. These findings were obtained using trypan blue exclusion test of cell viability in vivo and MTT test or clonogenic cell survival assay in vitro [9,26,27,28]. However, mechanisms underlying the effect of hyperthermia are poorly defined. In particular, these include understanding of the differences between in vitro and in vivo activities, a full potential of multimodal approach in tumor therapy by combining chemotherapy and hyperthermia, mutual interactions with chemotherapeutics, as well as advantages and disadvantages of different models in studying effects of hyperthermia. In our previous study, we investigated possible cytotoxic effects of combined chemoimmunotherapy and hyperthermic intraperitoneal chemotherapy (HIPEC). The focus was on direct and indirect cytotoxic and genotoxic effects and the role of different reparatory and immune system mechanisms. The research was performed using the mammary carcinoma cells (MCa) and EAT cells in vivo. Ehrlich ascites tumor is a suitable transplantable tumor model for studying antitumorous, angiogenic, and anti-inflammatory effects of different drugs and natural compounds. More precisely, this model demonstrates a direct interplay between the cells of immune system, angiogenesis and local tumor growth control [14].

It is known that hyperthermal treatment can enhance the cytotoxicity of some chemotherapeutic agents including CP, bleomycin, cyclophosphamide, mitomycin C, doxorubicin, and l,3-bis (2-chloroethyl)-nitrosourea [9,11,19,29].

HIPEC treatment may be enhanced with propolis and various flavonoid compounds such as QU, which are used as sensitizers to increase the effect of hyperthermia and reduce kidney and liver damage caused by cytostatics [9,28]. Preventive application of QU has shown a beneficial and synergistic effect as QU acted as sensitizer to hyperthermal treatment in many tumors [9,23,24,27]. However, the therapeutic potential of QU is quite unknown. According to Li et al. [25], QU at low concentrations reduced the therapeutic effects of CP and other anti-neoplastic drugs in ovarian cancer cells in vitro. In this study, we investigated the ability of QU to act as sensitizer and to enhance effects of hyperthermia and CP on bladder cancer cells in vitro and EAT cells in vivo.

Our data indicate that hyperthermia alone has a limited effect on T24 and UMUC cells in in vitro conditions. QU at a concentration of 50 µM sensitized these tumor cells in hyperthermia and significantly reduced the viability and clonogenicity (Figure 1 and Figure 2). This is consistent with the results obtained in melanoma, colon and HeLa cells, and human acute leukemia blast cells [23,28]. Conversely, QU in combination with CP did not additionally sensitize T24 and UMUC cells to heat as revealed by MTT assay in both cell lines. T24 cells are more sensitive to CP and a higher concentration of QU compared to UMUC cells (Figure 1). However, no statistical (*p* ˃ 0.05) difference was observed between the respective groups under physiological and hyperthermic conditions. QU and CP administered at higher concentration (50 µM) caused a lethal effect on T24 cells leading to inability to form colonies, while CP at higher concentration had a lethal effect on UMUC cells (Figure 2). However, the clonogenic assay showed that treatment of T24 and UMUC cells with high concentrations of QU or CP, as well as their combination, induced significant cell damage that disrupted cell division and colony formation (Figure 2), possibly due to a higher rate of CP intercalation into the DNA. The increased incorporation of CP into the DNA of the T24 cells was also shown by the lower electrophoretic mobility of the DNA in the agarose gel using a comet assay. Using a comet assay, we observed that the combination of QU and CP at both concentrations under physiological condition reduced the tail length and the amount of DNA in relative to the QU (data not shown). The same observations are described in several papers [9,11,16,23,24,27,30] with other antioxidants such as vitamin A, C, and E or other flavonoids where the authors suggest that free radicals may participate in the formation of CP-induced DNA-DNA or DNA-protein crosslinks. Under hyperthermic conditions, DNA damage was considerable and difficult to detect.

Obviously, the results of the MTT test, a colorimetric assay for assessing cellular metabolic activity, is less conclusive then the colony formation assay that is based on the ability of a single cell to form a colony. Thus, according to the results of clonogenic assay which is the method of choice for determining cell reproductive death and effectiveness of cytotoxic compounds [31], it seems that combination of QU and CP can be promising approach in therapy of bladder cancer. Short-term exposure of T24 cells and UMUC cells to QU and CP, which mimics clinical intravesical therapy, may be beneficial for bladder cancer treatment under physiological and hyperthermic conditions. Translation of these facts into in vivo model, may mean, that the local concentration of CP in the bladder would be significantly higher without parallel increase of CP in the serum. In addition to reducing systemic cytotoxicity, hyperthermia may increase chemotherapeutic uptake, and further influences the intracellular distribution and metabolism of drugs, and/or inhibit repair of DNA damage in bladder cancer cells. All these effects lead to an improvement of the effect of chemotherapeutic agents [8,9,10,11]. Furthermore, QU shows a selective effect; protects normal cells and inhibit proliferation and colony formation of bladder cells [13,32]. Furthermore, we also demonstrated that combined treatment with QU or CP and hyperthermia may have an additive, synergistic or lethal effect in vitro (Table 1 and Table 2).

In vivo, this multimodal approach exerted the synergistic effect under both temperature related conditions (IC = 1.826 or 2.363 and IC = 1.660 or 1.857 at dose CP5 or CP10) (Table 5). It seems that additional factors contributed to an increased antitumorogenic effect in vivo, which was manifested through reduced number of cells in the abdominal cavity (Figure 3), and significantly increased survival of the mice (Figure 4 and Table 3). Hyperthermia alone can induce genotoxic effects in vitro and in vivo such as sister chromatid exchanges chromosome aberrations and micronuclei, apoptosis, necrosis, and may damage the plasma membrane and inactivate heat-labile proteins, which lead to protein denaturation and subsequent aggregation [9,11,16,19]. These effects can change the number of cells in the abdominal cavity leading to increased survival rate of animals (Figure 4 and Table 3). On the other hand, hyperthermia treatment increases cytotoxic effect of CP by inducing structural and functional changes in cells. According to obtained results from this study, as well as from our previous data and the data of other authors, treatment with hyperthermia and CP leads to cellular changes and a loss of cellular homeostasis [9,23,33] through different mechanisms: (1) increased accumulation of CP; (2) protein denaturation and aggregation, with consequential cell cycle arrest; (3) disruption of mitotic spindle and enzymes; (4) inactivation of protein synthesis, inhibition of DNA synthesis, transcription, RNA processing and translation; (5) enhanced adduct formation with critical structures; (6) occurrence of different lesions at hyperthermic temperatures and heat-induced inhibition of DNA repair processes; (7) increased degradation of aggregated/misfolded proteins through proteasomal and lysosomal pathways; (8) disruption of the membrane cytoskeleton; (9) effects on tumor oxygenation and tumor micro-environment (e.g., uncoupling of oxidative phosphorylation) that lead to decreased levels of ATP; and (10) alterations in membrane permeability that cause increases in intracellular levels of Na^+^, H^+^, and Ca^2+^ (see [9,16]). All of these mechanisms, together with increased active or passive transport across the damaged cell membranes and increased CP solubility and interactions with DNA to form DNA adducts, primarily intra-strand crosslink adducts, contribute to intracellular CP accumulation which culminate in the activation of apoptosis, reduced cell number and increased life span of animals (Table 2 and Figure 1) [9,23,24,27].

Unlike in vitro studies, the in vivo model has an additional advantage, as it depends on various elements of the immune system, thus increasing immune surveillance and offering protection against tumor growth. This model also includes additional mechanisms such as antiestrogenic, antiangiogenic, and pro-apoptotic/necrotic mechanisms, selective destruction of tumor cells in hypoxic and low pH environments, and ability to reverse resistance to certain chemotherapeutic drugs [16,20,28]. According to this and ours previous and literature data, QU interferes with CP by acting on tumor cells through several mechanisms including: (1) increased immunity and direct DNA damage induced by apoptotic processing; (2) microenvironment remodeling to enhance the accumulation and penetration of CP into the tumor site; (3) altered metabolic activity of CP, acting as hyperthermic sensitizer by inducing intracellular lactate accumulation and acidification (increasing lysosomal activity with low pH and heat-induced cell membrane damage); (4) QU also triggered apoptosis and cell cycle arrest in cancer cells to sensitize them to CP chemotherapy; (5) QU enhanced ROS production in tumor cells to activate the intrinsic pathway of apoptosis through the induction of mitochondrial dysfunction and down-regulated anti-apoptotic factors such as Bcl-2 and Bcl-XL while it upregulated apoptotic factor Bax and Bid; (6) QU reversed multidrug resistance; it targets multidrug resistance proteins (MRPs) to inhibit the efflux of CP from cancer cells; (7) QU was capable inhibit HSP27, HSP70, HSP72, and HSP90 and stimulate apoptosis through induction of ER stress and inhibits STAT3 phosphorylation; (8) inhibited angiogenesis; (9) different mechanism which lead to synergistic action QU and CP (QU act on topoisomerase I and II, interact with estrogen binding sites type II and, inhibit different signal pathways involved in cell proliferation while CP act on the formation of DNA adducts and ROS; (10) MiRs, NF-κB, MAPK, AMPK, and HIF are also molecular pathways that are affected by QU with consequential enhancement of the CP chemotherapy; and (11) inhibition of protein kinase δ that enhances cytotoxic effects with CP and hyperthermia while protect kidney cells [9,12,13,16,19,20,21]. Thus, QU may be included in providing protection against CP mediated renal and liver injury: (1) QU reduces vascular permeability, decreases plasma volume loss after thermal injury and (2) protects against RBC deformity and platelet aggregation, and stabilizes lysosomal membranes [9,16,22,23,24,25,26,27]. Antioxidative and antiperoxidative properties of QU, based on free radical scavenger activity, increases the activity of superoxide dismutase, catalase, and glutathione in healthy cells [9,16].

We have found that QU increased effect of CP only at lower dose of CP (5 mg kg^−1^), while higher dose of CP had slightly lower effect as compared to CP10 alone under either condition. Furthermore, treatment of mice with QU alone was ineffective in the advanced stage of tumor growth and the ILS% was negative compared to the control. In general, hyperthermia at 43 °C mainly enhanced the survival of mice when compare to physiological condition. The differences between the ILS% values at 37 and 43 °C were as follows (%): control, 19.88; QU, −15.04; CP5, 104.30; CP5 + QU, 57.52; CP10, 62.36, and CP10 + QU, −6.48, respectively. Despite the increase in survival rate, there was no statistically significant difference in survival between the corresponding groups treated under physiological conditions compared to hyperthermic conditions, except for hyperthermal treatment alone or in combination treatment with cisplatin at a dose of 5 mg kg^−1^ while QU treatment had a negative effect.

According to this data, it is apparent that higher dose of CP greatly increases the CP’s toxicity under hyperthermic conditions, which is consistent with our previous results regarding the number of micronuclei in peripheral blood reticulocytes in vivo [28]. Specifically, a lower dose of CP with hyperthermal treatment achieved synergistic anticancer activity and reduced CP toxicity in liver, kidney, and other tissues and organs. This data indicate that under hyperthermic conditions CP alone, or combined with QU, may be the treatment of choice that resembles intraoperative hyperthermic intraperitoneal chemotherapy (HIPEC) or clinical intravesical therapy.

Interestingly, the number of mononuclear leukocyte cells was significantly increased in peripheral bloodstream after treatment with CP, especially at 37 °C, in relation to polymorphonuclear cells. The ratio between mononuclear and polymorphonuclear cells changed in favor of mononuclear leukocyte cells (macrophages and lymphocytes), while the treatment with QU after CP increased the number of neutrophils in the peripheral blood. 

The number of active macrophages was also increased in the peritoneal cavity at 43 °C as indicated by the macrophage spreading index (Table 6). It is possible that an increase of infiltrating monocytes and macrophages in the tumors may be important factor in tumor regression and enhanced survival of the mice [9,34,35,36]. Tumor regression may be mediated by a complex interaction between the innate and adaptive immunity. The innate mechanism is based on soluble and cellular components that trigger inflammatory events in tumor microenvironment and cytokine production that stimulates dendritic cells and enhances the capacity of peritoneal macrophages to kill tumor cells [9,36]. This can be achieved via direct cytotoxic effect or via an indirect cytotoxic activity by “preparing” tumor cells to their own elimination by immune cells such as natural killer (NK) cells or cytotoxic T lymphocytes using a Fast or TNF-related apoptosis-inducing ligand (TRAIL)-dependent pathway [37]. Furthermore, chemotherapy together with hyperthermia can modify interaction between tumor and dendritic cells, enhance maturation of dendritic cells, and enhance endocytosis of tumor cells by dendritic cells as well as increase the presentation of tumor antigens to T cells, especially by absorption of neoplastic tumor fragments after tumor cell destruction with HIPEC treatment [37,38,39]. In addition, HIPEC treatment increases the sensitivity of tumor cells to activate antitumor effector cells. Thus, hyperthermia may have a direct suppressive effect on tumor cells, or it may act indirectly via enhanced host immune function [9].

After heat stress, the heat shock proteins (HSPs) can activate tumor immunity by increasing antigen presentation through CD91 receptors, or via toll-like receptors (TLRs). In addition, tumor cells are involved in the production of “danger signals” that are required for dendritic cell maturation and tumor antigen presentation to T cells [40,41]. Some authors have also shown that intraperitoneal application of chemotherapeutic agents such as anthracycline (doxorubicin), mitomycin C, or CP may enhance the capacity of peritoneal macrophages to destroy tumor cells [37,38,39]. The underlying mechanisms of CP chemotherapy in advanced tumor stage may be: (1) overcoming the immunosuppression induced by the regulatory T cells (Treg) and myeloid derived suppressor cells (MDSC); (2) enhancing the maturation of dendritic cells and their function by displaying tumor antigens; (3) recruitment and proliferation of effector cells and strengthening the activity of cytotoxic T cells; (4) enhancing the penetration of immune cells into the tumor nucleus; (5) upregulation of MHC class I expression; (6) upregulation of the lytic activity of cytotoxic effectors; and (7) treatment of the cells by anticancer drugs at the temperature of 43 °C that increase tumor cell sensitivity to activate lymphocytes [9,37,38,39].

According to the mentioned data, the immunogenic effect of hyperthermia together with cisplatin can significantly increase therapeutic efficacy to tumor cells with low normal tissue damage leading to long-term tumor regression and increased animal survival (Figure 4 and Table 4 and Table 6). 

EAT is a rapidly growing tumor with aggressive behavior, meaning that the innate immune response is important for controlling its growth, especially the inflammatory response. Thus, the neutrophilic inflammatory response is essential for control of Ehrlich tumor growth. Activated neutrophils may induce tumor destruction by releasing a variety of factors (cytokines, enzymes, chlorinated oxidants, etc.) that are involved in direct tumor destruction, extracellular lysis, inhibition of angiogenesis and activation of other reactive cells, resulting in NK cell, T cell, and antibody-dependent cytotoxicity. However, the high influx of these cells promotes tumor development [42] as observed in control and QU groups (Figure 6). This effect is probably related to angiogenesis and releasing of growing factors induced by inflammation that are necessary for tumor development. However, it should be noted that macrophages can exert opposite effects, antitumor as well as pro-tumor activity. In addition to the macrophage-mediated bystander effect initiated by tumor cell apoptosis and the facilitation of antigen recognition, such as phosphatidylserine on the cell surface and enhanced killing of non-apoptotic bystander tumor cells, there are also different tumor-promoting functions of macrophages after chemotherapy that can misdirect macrophage-orchestrated tissue repair response resulting in chemo resistance [43]. According to that, it seems that chemotherapeutics-induced reduction of the macrophage infiltration into tumors may help in avoiding the pro-tumor activity of macrophages. Therefore, it is very important to investigate the role of macrophage and neutrophils in tumor growth and angiogenesis in relation to tumor microenvironment that affects their polarization and leads to the breakdown of phagocytic and tumoricidal activity of macrophage to pro-tumor activity. It is possible that CP plays a role in immunomodulation, specifically by altering the ratio of M2/M1-like macrophages [44,45] and in this way can achieve not only the therapeutic synergism, but also a long-term immunity against tumor growth, leading to increased survival of the animal (Figure 6).

Furthermore, we have noted the improvement of hepatorenal function after 20 days in relation to the control group and mice treatment only with QU, which is consistent with the increased survival of animals after treatment with CP or combination of CP and QU (Figure 6).

In conclusion, our results suggest that combined treatment with CP and QU may increase the death of tumor cells under physiological and hyperthermic conditions and could be of clinical relevance in locoregional chemotherapy without systemic toxicity. According to our results, QU in combination with a lower dose of CP in vivo was more effective in anti-cancer therapy than in vitro treatment, especially under hyperthermic condition. HIPEC treatment probably induces local inflammation and recruits effectors of antitumor immunity in vivo, thus increasing cytotoxic effect of CP and resulting in a long-term survival benefit for animals with EAT. The advantages of HIPEC or bladder intracavitary hyperthermic chemotherapy over single chemotherapy can be attributed to the synergy of local hyperthermia treatment, chemotherapy and immunomodulation. It appears that QU could increase sensitivity of chemotherapy and reduce toxicity to healthy cells. However, it should be noted that we can’t exclude the possible role of QU in reducing the toxicity of CP in therapeutic treatment to tumor cells due to possibility of collecting reactive radicals. Moreover, according to the obtained results, further studies are needed to examine the in vivo effects co-administration of quercetin with cisplatin and, the complex network of interactions between macrophages, neutrophils and the activation of effective adaptive responses and chemotherapy treatment in different contexts, including different microenvironment, pH, inflammation, cytokines, tumor immunogenicity, tissue of origin, characteristics of different drugs, dose as well as tumor types.

## 4. Material and Methods

### 4.1. Materials

Quercetin dihidydrate (QU, 98% purity) was purchased from Fluka, BioChemica, Switzerland. The anticancer drug cisplatin [*cis*-diamindikloroplatinum(II)] was supplied by Pliva, Zagreb, Croatia. F-12 Coon’s Modification medium, MTT [3-(4,5-dimethylthiazol2-yl)-2,5-diphenyltetrazolium bromide] for the evaluation of cell viability and proliferation, and fetal calf serum (FCS) were purchased from Sigma-Aldrich, St. Louis, MO, USA.

### 4.2. In Vitro Experiment

#### 4.2.1. Cells and Culture Conditions

Human bladder cancer cell lines T24 and UMUC2 were used and cultured in F-12 Coon’s modification medium supplemented with 10% heat-inactivated FCS, 100 U mL^−1^ penicillin and 100 µg mL^−1^ streptomycin (complete medium) in a humidified atmosphere of 95% air and 5% CO_2_ in incubator at 37 °C. Cells were allowed to attach for 24 h before the treatment.

#### 4.2.2. Cytotoxicity Assay

MTT assay was performed to determine the cytotoxicity of QU, CP or their combination on T24 and UMUC2 bladder cancer cells. T24 and UMUC2 were seeded at 1 × 10^4^ per well in 96-well culture plates and incubated overnight with F-12 Coon’s Modification medium containing 10% FCS. The cells were preincubated with 1 or 50 µM concentration of QU (QU1 or QU2) for 2 h at 37 °C [46,47]. According to our previous data and data from other authors, higher concentration of QU was selected because IC_50_ was between 50–100 µM [46,47,48]. After QU treatment, the cells were washed 3 times with phosphate-buffered saline (PBS) and incubated in fresh medium with or without 1 or 50 µM concentration of CP (CP1 or CP2) for 1 h [8,46,48]. Two concentrations of QU or CP (low and high) were taken to make easier monitoring the interaction between QU and CP and the contribution of each component to the cytotoxic effect. One group of cells was incubated at 37 °C in the presence or absence of CP, while the other group was exposed in another incubator at 43 °C. The percentage of survived cells was determined by 3-(4,5-dimethylthiazol2-yl)-2,5-diphenyltetrazolium bromide (MTT) assay. After incubation for the indicated time, MTT (20 μL of 5 mg mL^−1^) was added to each well and incubated at 37 °C for 4 h, after which the MTT solution in the medium was aspirated. To achieve the solubilization of the formazan crystals formed in viable cells, 150 μL DMSO was added to each well before the absorbance was measured at 570 nm using a microplate reader (Bio-Rad, Model 550, Tokyo, Japan). The effect of QU on growth inhibition was expressed as a percentage of cell viability where vehicle-treated cells were taken as 100% viable.

#### 4.2.3. Clonogenicity (Tumor Cell Survival) Assay

To mimic clinical intravesical therapy, approximately 200 cells of T24 or UMUC2 were plated in 5 mL complete medium onto 60 mm tissue culture dishes. Cells were allowed to attach for 24 h before the addition of 2.5 mL complete medium containing 1 or 50 µM QU. Dishes were placed in the incubator for 2 h, and then gently rinsed with PBS three times to remove the QU. Complete culture medium was added to each dish with or without CP for 1 h at concentration of 1 or 50 µM. One group of cells was incubated at 37 °C and the other at 43 °C in the presence or absence of CP. After 1 h cells were rinsed with PBS three times to remove the CP and dishes were returned to the incubator for up to 14 days until survived cancer cells formed sufficiently large colonies. Cultures were maintained in a humidified atmosphere of 5% CO_2_ at 37 °C. After 14 days colonies were fixed with 100% methanol, stained with Giemsa stain (5%) and the number of colonies per dish were counted. The colony is defined to consist of at least 50 cells. The assay was repeated three times in triplicate using each cell line. The plating efficiency (PE) was calculated as:

(1) $$PE=\left(\frac{Colonies\;formed}{Cells\;seeded}\right)\times \;100\%.$$

### 4.3. In Vivo Experiment

#### 4.3.1. Animals

Female Swiss albino inbred mice, weighing 20–25 g, approximately 2 months old, obtained from the Department of Animal Physiology, Faculty of Science, University of Zagreb, were used in the study. Animals were housed up to 5 per cage and were maintained on a pellet diet (Standard Diet 4RF 21 GLP certificate, Mucedola, Italy) and water ad libitum. They were kept under a 12 h light, 12 h dark cycle at 60% humidity. Each experimental group contained 15 mice. Animal studies were performed in compliance with the Guidelines of the Republic of Croatia (Law on the Welfare of Animals, NN 102/2017; Regulations for the Environmental Conditions of Experimental Animals, Special Conditions for the Facilities and Experiment Categories, N.N. #176, 2004) and according to the Guide for the Care and Use of Laboratory Animals, Department of Health and Human Services, DHHS Publ. (National Institutes of Health, NIH) 86-123, 1985. All the guidelines enforced in Croatia are in accordance with the internationally accepted principles for laboratory animal use and care, as found in the European Community guidelines (EEC Directive of 1986; 86/609/EEC). The ethical committee (Faculty of Science, University of Zagreb, Croatia) approved the study (approval code: 251-61-01/139-09-2, date of approval: 5 December 2019).

#### 4.3.2. Tumor Cells

Ehrlich ascites tumor (EAT) is highly transplantable, undifferentiated and fast growing malignant tumor which originally developed as a spontaneous breast carcinoma in a mouse. EAT cells were maintained in female Swiss albino mice in ascitic form by serial intraperitoneal transplantations at 7- or 9-days intervals. After harvesting and preparation of cells, their total number and viability were determined in Bürker-Türk counting chamber using trypan blue dye. The desired concentration of tumor cells (2 × 10^6^ cells per 0.5 mL) was obtained by dilution with saline (0.9% sodium chloride solution).

#### 4.3.3. Hyperthermal Treatment

Intraperitoneal hyperthermia was induced by *i.p.* administration of 2 + 2 mL saline solution preheated in water bath to 43 °C and separated by 5 min [9]. Hyperthermia treatment (HT) was performed immediately before the *i.p.* application of CP. CP was administered intraperitoneally at dose of 5 or 10 mg kg^−1^. Systemic body temperature was determined before and during hyperthermia procedure using an electronic thermometer (BAT-10, Physitemp Instruments, Clifton, NJ, USA) with a rectal probe (RET-3, Physitemp Instruments, Clifton, NJ, USA). Intraperitoneal temperature was measured by the needle probe that was introduced to a depth of 1 cm into peritoneal cavity of mice treated with hyperthermia. Hyperthermia with applied procedure was well tolerated by the animals.

It is important to emphasize that during the heating phase, the intra-abdominal temperature was stable, ranging from 42.5–43 °C, whereas rectal temperature was not significantly affected. During the cooling phase, the temperature in the peritoneal cavity of treated mice decreased to normal values approximately 15 min after applying the hyperthermal procedure [11,19].

#### 4.3.4. Experimental Procedure

A total of 180 mice were used in the study. All mice were inoculated *i.p.* with 2 × 10^6^ EAT cells in 0.5 mL of Hank’s balanced salt solution (HBBS) on day 0. Mice were divided into 12 experimental groups. CP treatment (5 or 10 mg kg^−1^*, i.p.*) was initiated on day 5 of tumor transplantation in the advanced stage of tumor progression when the cells enter into exponential growth period. Treatment with CP is performed under physiological (37 °C) and hyperthermic (43 °C) conditions. This procedure was performed in all hyperthermia groups regardless of CP administration. Mice in all physiological groups were subjected to the same procedure, except the saline was preheated to 37 °C in water bath. Intraperitoneal treatment with QU at a dose of 50 mg kg^−1^ was initiated on day 6 and 12 after inoculation with EAT cells. QU was prepared in ethanol and further dilutions were made in water. The final concentration of ethanol was less than or equal to 0.1%. Ethanol (0.1%) was also used in the control group. No difference between water as a control and 0.1% of ethanol in water was observed in preliminary experiments.

One part of the animals was sacrificed on day 13 after tumor cell inoculation (*n* = 6 per each group) for the analysis of total cell number in peritoneal cavity, spreading activity of macrophages, differential count of the cells present in the peritoneal cavity, and analysis of hematological and biochemical parameters from blood samples. The rest of the animals was kept for the monitoring animal survival after treatment.

#### 4.3.5. Survival Analysis

Life span of animals was evaluated by monitoring spontaneous death or by selective killing of animals showing signs of pain or suffering according to established criteria. Long surviving mice were euthanized on the 90th day by an overdose of anesthetic Narketan^®^10 (Vetoquinol, Lure, France) at a dose of 100 mg kg^−^^1^ and Xylapan^®^ (Vetoquinol Biowet, Gorzow, Poland) at a dose 5 mg kg^−^^1^ and cervical dislocation. Results were expressed as a percent of mean survival time of treated animals over mean survival time of the control group (treated vs. control, T/C%). The percentage of increase in life span (ILS%) was calculated by formula: ILS% = (T - C) / C × 100 where T represents mean survival time of treated animals and C the mean survival time of the control group. Long-term survivors (LTS%) were mice surviving more than 90 days after *i.p.* implantation of EAT cells. According to the criteria of the National Cancer Institute, T/C above 125% and ILS above 25% indicate that treatment had significant antitumor effect.

#### 4.3.6. Tumor Evaluation 

Viability of tumor cells was determined by using Bürker-Türk counting chamber, trypan blue dye and phase contrast microscopy. The assay is based on the ability of viable cells to exclude trypan blue dye and appear bright, whereas dead cells are stained blue. The total number of tumor cells present in the peritoneal fluid of each animal was counted and percent of growth inhibition was calculated as follows:

(2) $$\%\mathrm{Tumor}\;\mathrm{inhibition}\;\left(\mathrm{TI}\%\right)=\frac{\left(Av.num.of\;cells\;in\;control\;group-Av.num.of\;cells\;in\;exp.group\right)\times 100}{Av.num.of\;cells\;in\;control\;group}$$

where *Av. num.* is average number.

#### 4.3.7. Differential Count of the Cells Present in the Intraperitoneal Cavity

The cells in the peritoneal cavity of mice were harvested, stained with May Grünwald and water solution of Giemsa (1 part of Giemsa: 2 parts of water) and classified as macrophages, lymphocytes, neutrophils or tumor cells [49]. Differential cell counts were performed by counting at least 800 cells in each sample.

#### 4.3.8. Analysis of Macrophages Spreading in the Intraperitoneal Cavity

Functional activity of macrophages in the peritoneal cavity was determined by the spreading technique [50]. Briefly, 10^3^ cells in 0.1 mL of the cellular suspension obtained from the peritoneal cavity was placed over glass cover slips at room temperature for 15 min. The non-adherent cells were removed by washing with PBS, whereas the adherent cells were incubated in culture medium 199 containing 10 mM HEPES buffer at 37 °C for 1 h. Following incubation, the culture medium was removed and the cells were fixed with 2.5% glutaraldehyde for 10 min. Afterwards, the cells were stained with 5% Giemsa solution and examined under microscope. The percentage of spread cells was determined under 400× magnification. Spread cells were those that exhibited cytoplasmic projections, while the non-spread cells were rounded. Using an ocular grid, 200 macrophages were scored as either round or spread. An index of macrophage spreading (SI) was then calculated for each monolayer of each glass cover slip, as follows:

(3) $$\mathrm{SI}\;=\frac{\left(number\;of\;spreading\;macrophages\;\times \;100\right)}{200\;adherent\;cells}$$

i.e., SI—% of spreading macrophages.

#### 4.3.9. Analysis of Hematological and Biochemical Parameters

Blood samples were collected in separator tubes for serum collections. Serum was analyzed for several biochemical parameters including aspartate aminotransferase (AST), alanine aminotransferase (ALT), alkaline phosphatase (ALP), urea, creatinine, blood glucose levels (glucose), lactate dehydrogenase (LDH), total protein and serum calcium (Ca), and phosphate (P) levels using Alcylon 300 (Abbott, Chicago, IL, USA).

The evaluation of the hematological parameters was performed by using standard laboratory methods and blood cell counter Cell-Dyn^®^ 3700 (Abbott, Abbott Park, IL, USA). The number of erythrocytes (E), the average cellular volume of erythrocytes (MCV), haemoglobin (Hgb), haematocrit (Hct), mean cell haemoglobin (MCH), mean cell haemoglobin concentration (MCHC), total leukocyte count (L), and the total number of platelets (Plt) was determined.

#### 4.3.10. Polymorphonuclear/Mononuclear Leukocyte Ratio—P/M Activity

P/M activity was used for the evaluation of the immunopotentiating effect of QU. The ratio of polymorph nuclear to mononuclear leukocytes was taken as an indicator of the immunopotentiating capability. Blood samples were collected from tail veins of experimental and control mice on day 13 or 20 after inoculation of EAT cells. Differential counts of leukocytes were performed on blood samples using an automatic cell counter. 

#### 4.3.11. Estimation of the Interaction between QU, CP, and HT In Vitro and In Vivo Models

To evaluate the combined effect of CP and QU, T24 and UMUC2 cells were treated with different concentrations of CP and QU, alone or in combination, and cell viability was determined as explained in above Section 4.2.2, Section 4.2.3 and Section 4.3.2 (MTT assay, clonogenisity assay, and trypan blue cells viability assay). Two concentrations of QU or CP (low and high) were taken to make easier monitoring the interaction between QU and CP and the contribution of each component to the cytotoxic effect. The inhibitory effects of QU, CP and HT were calculated using the percentages of cells in treated (T) versus untreated cells (T/C). The combined effects of QU or CP and HT (combination index) was calculated using the formula %AB/(%A × %B), where A and B are the effects of each individual agent and AB is the effect of the combination. When the ratio is 1, the effect is considered additive. When the ratio is significantly greater than additive (> 1), it is termed synergistic. If it is significantly less than additive (< 1), then it is termed antagonistic according to Piantelli et al. [26,51]. Exactly, to calculate additive and synergistic effects we used the definitions listed in the Table 7.

### 4.4. Statistical Analysis

The experiments were performed in triplicate. The results were expressed as mean ± SD from three independent experiments. All data were analyzed by nonparametric Kruskal–Wallis test. Further analysis of the differences between the groups was made with multiple comparisons of mean ranks for all groups. Statistical analyses were performed using STATISTICA 13 software (StatSoft, Tulsa, OK, USA). The data were considered significant at *p* < 0.05. Treatment-dose specific survival curves were calculated by the Kaplan–Meier method [52], and comparison between the survival curves was made by log-rank test (α = 5%) [53].

## Figures and Tables

**Table 1:** Combination index values of the interactions between the inhibitory effect of quercetin and cisplatin under physiological and hyperthermic conditions on the survival of T24 and UMUC cells.

Treatments	T24 Cells				UMUC Cells			
	Physiological Conditions		Hyperthermic Conditions		Physiological Conditions		Hyperthermic Conditions	
	Combination Index	*p*	Combination Index	*p*	Combination Index	*p*	Combination Index	*p*
QU1CP1	1.230 ± 0.16	NS	1.640 ± 0.14	0.001	1.410 ± 0.28	0.01	1.618 ± 0.13	0.001
QU2CP1	1.388 ± 0.09	0.01	1.435 ± 0.20	0.001	1.495 ± 0.15	0.001	1.990 ± 0.14	0.001
QU1CP2	1.071 ± 0.13	0.001	1.150 ± 0.09	0.001	1.247 ± 0.07	0.001	1.656 ± 0.13	0.001
QU2CP2	1.392 ± 0.07	0.001	1.511± 0.11	0.001	1.180 ± 0.13	0.001	1.923 ± 0.15	0.001

The analysis was made according to the data represented in Figure 1. The combination index was calculated as described in Materials and methods. The combined effects of QU and CP treatment under physiological and hyperthermic conditions (combination index) was calculated using the formula %AB/(%A × %B), where A and B are the effects of each individual agent and AB is the effect of the combination. *p*, statistical significance values of the combination indices (AB) compared with the additive combination index [AB/(A × B)], nonparametric Kruskal–Wallis test, *p* < 0.05; *p* < 0.01; *p* < 0.001. Results are reported as the mean ± SD of three independently performed experiments. Abbreviation: QU1 or QU2—treatments with quercetin at concentrations of 1 or 50 µM; CP1 or CP2—treatments with cisplatin at concentrations of 1 or 50 µM; NS—not significant.

**Table 2:** Combination index values of the interactions between the inhibitory effect of quercetin and cisplatin under physiological and hyperthermic conditions on colony formation of T24 and UMUC cells.

Treatments	T24 Cells				UMUC Cells			
	Physiological Conditions		Hyperthermic Conditions		Physiological Conditions		Hyperthermic Conditions	
	Combination Index	*p*	Combination Index	*p*	Combination Index	*p*	Combination Index	*p*
QU1CP1	0.930 ± 0.08	0.001	0.898 ± 0.14	0.001	1.060 ± 0.15	NS	1.042 ± 0.09	0.001
QU2CP1	LE	-	LE	-	0.917 ± 0.13	NS	1.040 ± 0.06	0.001
QU1CP2	LE	-	LE	-	LE	-	LE	LE
QU2CP2	LE	-	LE	-	LE	-	LE	LE

The analysis was made according to the data from Figure 2. The combination index was calculated as described in Materials and methods. The combined effects of QU/CP treatment and hyperthermia (combination index) was calculated using the formula %AB/(%A × %B), where A and B are the effects of each individual agent and AB is the effect of the combination. *p*, statistical significance values of the combination indices (AB) compared with the additive combination index [(AB/A × B)], nonparametric Kruskal–Wallis test, *p* < 0.05. Results are reported as the mean ± SD of three independently performed experiments. Abbreviation: QU1 or QU2—treatments with quercetin at concentrations of 1 or 50 µM; CP1 or CP2—treatments with cisplatin at concentrations of 1 or 50 µM; LE—lethal effect; NS—not significant.

**Table 3:** Survival analysis of Swiss albino mice bearing Ehrlich ascites tumor after therapeutic treatment with cisplatin and QU under physiological and hyperthermic conditions.

Experimental Groups ^a^	Physiological Intraperitoneal Temperature (37 °C)				Intraperitoneal Hyperthermia (43 °C)			
	Mean Survival Time in Days (Range)	T/C% ^b^	ILS% ^c^	Long-Term Survivors (LTS%)	Mean Survival Time in Days (Range)	T/C% ^b^	ILS% ^c^	Long-Term Survivors (LTS%)
Control (EAT)	20.67 (19–22)	–	–	0	24.78 (24–28) ^ΔΔΔ^	119.88	19.88	0
QU	19.78 (18–21)	95.69	−4.31	0	16.67 (14–18) * ^ΔΔΔ^	80.65	−19.3517	0
CP 5 mg kg^−1^	33.00 (19–90) *	159.65	59.65	1 (11%)	54.56 (21–90) * ^Δ^	263.96	163.95	3 (33.33%)
CP 5 mg kg^−1^ + QU	67.00 (31–90) *	324.141	224.14	5 (55.55%)	78.89 (38–90) *	381.66	281.36	6 (66.66%)
CP 10 mg kg^−1^	76.33 (17–90) *	369.28	269.28	7 (77.77%)	89.22 (83–90) *	431.64	331.64	8 (88.88%)
CP 10 mg kg^−1^ + QU	80.78 (51–90) *	390.81	290.81	6 (66.66%)	79.44 (40–90) *	384.33	284.32	7 (77.77%)

^a^ Mice were treated with CP (5 or 10 mg kg^−1^, *i.p.*) 5 days after *i.p.* inoculation of EAT (2 × 10^6^/mouse) cells under physiological and hyperthermic conditions and with QU (*i.p.* 50 mg kg^−1^) 6 and 12 day after *i.p.* inoculation of EAT cells. The results are expressed as mean value of each experimental group (*n* = 9). ^b^ T/C% = T/C × 100; T—mean survival days of treated group; C—mean survival days of control group at 37 °C. ^c^ ILS% (increased life span %) = (T–C)/C × 100. According to the criteria of the National Cancer Institute, T/C above 125% and ILS above 25% indicate that treatment had significant antitumor effect. * Significantly different (*p* < 0.05; log-rank test) in relation to corresponding control at 37 or 43 °C. ^Δ^ Significantly different (^Δ^ *p* < 0.05; ^ΔΔΔ^ *p* < 0.001; log-rank test) between physiological and hyperthermic condition. Abbreviation: EAT, Ehrlich ascites tumor; QU, treatment with quercetin at a dose of 50 mg kg^−1^; CP5 or CP10, treatments with cisplatin at doses of 5 or 10 mg kg^−1^.

**Table 4:** Kaplan–Meier survival analysis of Swiss albino mice bearing EAT tumor after treatment under physiological and hyperthermic conditions.

	Kaplan–Meier Analysis ^a^					
Experimental Groups	Physiological Conditions (37 °C)		Hyperthermic Conditions (43 °C)		37 vs. 43 °C	
	*p* Value	χ^2^	*p* Value	χ^2^	*p* Value	χ^2^
Control (EAT)	-		-		0.0001	16.42
QU	0.0346	4.466	0.0015	10.044	0.0004	12.611
CP 5 mg kg^−1^	0.0060	7.564	0.0004	12.578	0,0198	5.432
CP 5 mg kg^−1^ + QU	0.0001	16.420	<0.0001	19.854	0.473	0.514
CP 10 mg kg^−1^	0.0060	7.564	<0.0001	19.859	0.4929	0.470
CP 10 mg kg^−1^ + QU	0.0001	16.420	<0.0001	19.859	0.7560	0.096

^a^ Significantly different (*p* < 0.05; log-rank test) in relation to control at 37 or 43 °C and between physiological and hyperthermic conditions.

**Table 5:** Combination index values of the interactions between the inhibitory effect of quercetin and cisplatin under physiological and hyperthermic conditions on number of Ehrlich ascites tumor cells.

Quercetin/Cisplatin Combination	Physiological Conditions		Hyperthermic Conditions	
	Combination Index	*p*	Combination Index	*p*
CP5 + QU	1.826 ± 0.20	0.001	1.660 ± 0.23	0.001
CP10 + QU	2.363 ± 0.13	0.001	1.857 ± 0.31	0.001

**Table 6:** Macrophage spreading index in peritoneal cavity of Swiss albino mice bearing Ehrlich ascites tumor after therapeutic treatment with cisplatin and QU under physiological and hyperthermic condition.

Experimental Groups ^a^	Macrophage Spreading Index (%)	
	Physiological Condition (37 °C)	Hyperthermic Condition (43 °C)
Control (EAT)	31.33 ± 12.40	30.71 ± 18.16
QU	52.67 ± 8.78	69.63 ± 16.53 *
CP 5 mg kg^−1^	54.00 ± 6.78	56.75 ± 9.54
CP 5 mg kg^−1^ + QU	78.00 ± 3.54 *^◊^	91.38 ± 6.21 *^◊^
CP 10 mg kg^−1^	58.29 ± 13.78	55.00 ± 15.17
CP 10 mg kg^−1^ + QU	83.86 ± 7.69 *	74.75 ± 11.57 *

^a^ Mice were treated with CP (5 or 10 mg kg^−1^, *i.p.*) 5th day after *i.p.* inoculation of EAT (2 × 10^6^/mouse) cells under physiological and hyperthermic condition and with QU (*i.p.* 50 mg kg^−1^) 6 and 12 days after *i.p.* inoculation of EAT cells. Results are reported as the mean ± SD (*n* = 6). * Significantly different (nonparametric Kruskal–Wallis test, *p* < 0.05) from control group. ^◊^ Significantly different (nonparametric Kruskal–Wallis test, *p* < 0.05) from group treated with cisplatin 5 mg kg^−1^. Abbreviation: EAT—Ehrlich ascites tumor; QU—treatments with quercetin at dose of 50 mg kg^−1^; CP5 or CP10—treatments with cisplatin at dose of 5 or 10 mg kg^−1^.

**Table 7:** Definitions of Drug Combination Effects.

Effect:	Combination Index (CI)
Antagonistic	SF AB > [(SFA) × (SFB)]
Additive	SF AB = [(SFA) × (SFB)]
Synergistic	SF AB < [(SFA) × (SFB)]

SF: surviving fraction A, B: drugs, AB: drugs used in combination [51].
